# Evaluation of Serum MicroRNAs (miR-9-5p, miR-17-5p, and miR-148a-3p) as Potential Biomarkers of Breast Cancer

**DOI:** 10.1155/2022/9961412

**Published:** 2022-01-24

**Authors:** Xiaoqin Li, Xinyue Tang, Kunsong Li, Ling Lu

**Affiliations:** ^1^Department of Oncology, Guangdong Second Provincial General Hospital, Guangzhou 510220, China; ^2^Department of Pulmonary Medicine, Affiliated Tumor Hospital of Xinjiang Medical University, Urumqi, Xinjiang, 830011, China; ^3^Department of Gynecology, First Affiliated Hospital of Chengdu Medical College, Chengdu 610500, China

## Abstract

**Background:**

MicroRNAs (miRNAs) play important roles in the initiation and progression of cancers. The purpose of the present study was to evaluate the use of serum miRNA biomarkers in the early diagnosis of breast cancer.

**Methods:**

The expression levels of *miR-9-5p*, *miR-17-5p*, and *miR-148a-3p* were analyzed by quantitative reverse transcription-polymerase chain reaction in 49 patients with newly diagnosed breast cancer and 49 healthy controls. The associations between *miR-9-5p*, *miR-17-5p*, and *miR-148a-3p* levels and clinicopathological parameters were also analyzed. Regression analysis and sensitivity and specificity analyses were used to determine the diagnostic efficacy of the miRNAs.

**Results:**

Serum levels of *miR-9-*5p and *miR-148a-3p* were significantly higher in breast cancer patients than in healthy controls (both *P* < 0.05), but *miR-17-5p* levels were not different between the two groups (*P* = 0.996). Serum *miR-9-5p* levels were markedly higher in patients with human epidermal growth factor receptor 2- (HER2-) positive breast cancer than in those with HER2-negative breast cancer (*P* = 0.049). Serum levels of *miR-9-5p* and *miR-148a-3p* were positively correlated with the presence of breast cancer, and both miRNAs had high sensitivity and specificity for the diagnosis of breast cancer.

**Conclusions:**

These findings provide evidence that serum *miR-9-5p* and *miR-148a-3p* levels may be used as noninvasive biological markers for the clinical diagnosis of breast cancer.

## 1. Introduction

Breast cancer is one of the most common malignant cancers globally. It is the leading cause of cancer-specific deaths in women and the second leading cause of total deaths in women [1]. More than 270,000 new cases of breast cancer were confirmed in 2019 [2]. Although the prognosis of breast cancer patients has greatly improved, approximately 685,000 women died from this disease globally in 2020 [3]. Despite rapid developments in modern medical technology, the early diagnosis of breast cancer remains critical to improve the efficacy of treatments and the prognosis of this disease [4].

Sentinel lymph node biopsy, which has replaced axillary lymph node dissection, has been validated as the standard surgical procedure for the routine surgical management of breast cancer [5]. Similarly, molecular biomarkers, such as progesterone receptor (PR), estrogen receptor (ER), and human epidermal growth factor receptor 2 (HER2), have been used to improve the prognostic stratification and predict the response to treatments [6]. However, given that blood collection has the advantages of simplicity, convenience, and noninvasiveness, liquid biopsy is becoming an increasingly relevant diagnostic method for oncological diseases. Thus, it is necessary to identify more accurate and reliable biomarkers that can be noninvasively, conveniently, and reliably used for the early diagnosis of breast cancer. Hence, noninvasive biomarkers, particularly blood microRNAs (miRNAs), have been extensively studied recently.

miRNAs are a diverse group of small noncoding RNA molecules with a length of 18 to 25 nucleotides [7]. They play important roles in various physiological and pathological processes related to cell proliferation, apoptosis, differentiation, and invasion [8]. Recent studies have demonstrated that the levels of some miRNAs correlated with clinical and biological features of cancers, and they were aberrantly expressed in various types of cancers, such as lung cancer [9], certain myelomas [10], breast cancer [11], colorectal cancer [12], and hepatocellular carcinoma [13]. The evaluation of miRNA levels in blood, serum, or plasma may be helpful for tumor diagnosis [14].

miRNAs may participate in the development of breast cancer. *miR-9-5p* is highly expressed in breast cancer and is closely related to the biological behaviors of tumor cells, such as their promotion of angiogenesis, metastasis, and epithelial-mesenchymal transition [15, 16]. Studies have shown that *miR-17-5p* inhibited the proliferation and invasion of breast cancer cells. The low levels of *miR-17-5p* observed in the serum and cancer tissues of breast cancer patients are helpful for clinical diagnosis [17]. *miR-148a-3p* may be downregulated in the plasma of patients with breast cancer, and this is associated with lymph node metastasis [18].

The possibility of detecting cell-free miRNAs and the specific characteristics of miRNAs, such as tissue specificity and stability, indicates that miRNAs may be important biomarkers [19]. Although the serum level of *miR-17-5p* may be used as a marker for breast cancer recurrence [20], there are no previous studies showing the use of this biomarker in patients with newly diagnosed breast cancer. Some studies have found that *miR-148a-3p* was downregulated in breast cancer patients, but the authors of these studies did not further analyze the specificity or sensitivity of *miR-148a-3p* for the diagnosis of breast cancer [18].

In this study, we aimed to evaluate the levels of *miR-9-5p*, *miR-17-5p*, and *miR148a-3p* in serum samples from breast cancer patients, to analyze their relationships with clinicopathological parameters, and to preliminarily explore their diagnostic value for breast cancer.

## 2. Materials

### 2.1. Patients

In this study, a total of 49 patients with a new (within 3 months) diagnosis of primary breast cancer and 49 age-matched healthy women, who were referred to the Guangdong Second Provincial General Hospital from January 12, 2019, to December 21, 2020, were recruited. Patients were included in the study if they (1) had not received radiotherapy, chemotherapy, or surgical resection before enrollment, (2) had no history of smoking or drinking, and (3) showed no evidence of organ metastasis. Patients were excluded if they (1) had severe liver or kidney damage, (2) had other malignant tumors, (3) had an autoimmune or other disease, or (4) had incomplete clinical data. The healthy controls were volunteers with normal mammography and ultrasonography results and without a history of malignant disease.

All of the subjects or their families provided written informed consent before participating in the study. This study was approved by the Ethics Committee of Guangdong Second Provincial General Hospital.

We collected the baseline characteristics of breast cancer patients and controls, including age, body mass index (BMI), age at first pregnancy, menopausal status, use of estrogen, and family history of breast cancer. Breast cancer was histologically confirmed for all patients. Clinical characteristics were collected through interviewer-administered questionnaires and/or from medical records. ER, PR, and HER2 status were determined by immunohistochemistry.

Peripheral blood was collected in BD vacutainer tubes (BD Biosciences, Franklin Lakes, NJ, U.S.A.) from each subject at the time of diagnosis and before commencing any therapy. Serum was separated within 2 hours of blood sample collection and then stored in aliquots in Eppendorf tubes at -80°C until analysis.

## 3. Methods

### 3.1. RNA Isolation and Reverse Transcription-Polymerase Chain Reaction Assay

Total RNA was extracted from serum samples using a miRNeasy extraction kit (Qiagen, Hilden, Germany) following the manufacturer's instructions. The quality and quantity of the RNA samples were determined using a NanoDrop spectrophotometer (Thermo Fisher Scientific, Waltham, MA, U.S.A.). miRNA levels were determined by quantitative reverse transcription-polymerase chain reaction (qRT-PCR). A microRNA Reverse Transcription Kit (Applied Biosystems, Foster City, CA, U.S.A.) was used to reverse transcribe miRNA according to the manufacturer's guidelines. Reactions were performed in an ABI 7500 Real-Time PCR System (Applied Biosystems). The levels of *miR-9-5p*, *miR-17-3p*, and *miR-148a-3p* were normalized to *miR-16* levels using the Relative Expression Software Tool (REST version 2008).

### 3.2. Statistical Analysis

All analyses were performed using SPSS 22.0 software (SPSS Inc., Chicago, IL, U.S.A.). Normally distributed continuous variables were expressed as the mean values ± the standard errors, and Student's *t*-test was used to compare parametric data between groups. Categorical variables were expressed as percentages, and a chi-square test was used to compare classified variables between groups. Pearson's correlation was used to analyze the correlation between the level of miRNAs and the presence of breast cancer. Receiver operating characteristic curves were generated to evaluate the predictive values of the candidate miRNAs. A *P* value less than 0.05 was considered to be statistically significant.

## 4. Results

### 4.1. Baseline and Clinicopathological Characteristics of Breast Cancer Patients and Controls

The features of each group are summarized in Table 1. The mean ages of the patients with breast cancer and healthy control subjects were 55.6 ± 9.58 and 54.2 ± 8.77 years, respectively. No statistically significant differences were found in the baseline characteristics between the two groups for age, BMI, age at first pregnancy, menopausal status, use of estrogens, and family history of breast cancer (Table 1). The clinicopathological characteristics of the breast cancer patients are shown in Table 2.

### 4.2. Serum miRNA Levels in the Breast Cancer and Control Groups

**Figure 1:**
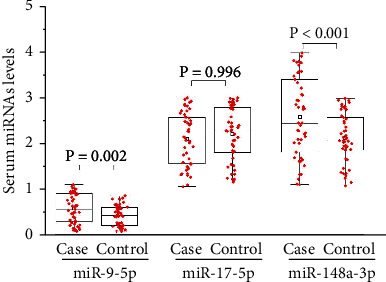
Serum levels of the three microRNA candidates in the case and control groups.

We compared the serum levels of *miR-9-5p*, *miR-17-5p*, and *miR-148a-3p* in the case and control groups. The levels of *miR-9-5p* and *miR-148a-3p* were significantly higher in the case group than those in the control group (*miR-9-5p*: 0.514 ± 0.327 vs. 0.356 ± 0.233, *P* = 0.002; *miR-148a-3p*: 2.557 ± 0.891 vs. 2.086 ± 0.552, *P* < 0.001), but no significant difference was found in the serum levels of *miR-17-5p* between the two groups (Table 3 and Figure 1).

### 4.3. Comparison of Serum miRNA Levels in Various Groups of Breast Cancer Patients

To examine the effect of menopausal status on the serum levels of miRNAs, we divided the case group into premenopause and postmenopause subgroups. The serum levels of *miR-9-5p* and *miR-148a-3p* were significantly higher in the postmenopause subgroup than those in the control group (*miR-9-5p*: 0.557 ± 0.326 vs. 0.356 ± 0.233, *P* = 0.001; *miR-148a-3p*: 2.609 ± 0.931 vs. 2.086 ± 0.552, *P* = 0.002). However, the levels of *miR-9-5p* and *miR-148a-3p* were not significantly different between the premenopause subgroup and the control group or between the pre- or postmenopause subgroups. Similarly, there was no significant difference in the levels of *miR-17-5p* between either the breast cancer subgroup and the control group or between the pre- or postmenopause breast cancer subgroups (Table 4).

To determine whether there were relationships between the serum levels of *miR-9-5p*, *miR-17-5p*, and *miR-148a-3p* and clinicopathological features, the breast cancer patients were stratified according to ER, PR, and HER2 status. There was no statistically significant difference in the serum levels of *miR-17-5p* between the different hormone status subgroups. The serum levels of *miR-9-5p* were significantly higher in the HR-positive patients than those in the HR-negative patients (0.577 ± 0.357 vs. 0.357 ± 0.25, *P* = 0.049). However, the serum levels of miR-9-5p were not significantly different between the HER2-positive patients and the control subjects. Furthermore, the levels of *miR-9-5p* and *miR-148a-3p* were significantly higher in the ER-positive, ER-negative, PR-positive, and PR-negative breast cancer patient subgroups than those in the control group (Table 4).

### 4.4. Regression Analysis of miRNA Levels

Regression analysis was performed to analyze the relationships between miRNA levels (*miR-9-5p*, *miR-17-5p*, and *miR148a-3p*) and breast cancer. The regression coefficient for *miR-9-5p* was 7.69 (95% confidence interval (CI): 5.88, 9, 79), which indicated a significant positive correlation with the presence of breast cancer (*P* < 0.001). The *miR-148a-3*p levels were also significantly positively correlated with the presence of breast cancer (regression coefficient, 2.48; 95% CI: 0.77, 4.20; *P* < 0.001; Table 5).

### 4.5. Diagnostic Value of Serum miRNA Levels in Breast Cancer

We calculated sensitivity and specificity values for the use of *miR-9-5p*, *miR-17-5p*, and *miR-148a-3p* levels in breast cancer diagnosis. Higher sensitivity and specificity were observed for *miR-9-5p* (85.2%; 95% CI: 76.3, 99.1 and 93.7%; 95% CI: 72.5, 98.7, respectively) and *miR-148a-3p* (86.6%; 95% CI: 70.4, 92.8 and 87.5%; 95% CI: 73.8, 93.9, respectively) than for *miR-17-5p* (Table 6).

## 5. Discussion

Breast cancer is one of the most common malignant tumors and its incidence is increasing every year. Moreover, it shows a younger median age at diagnosis [2], which brings great challenges for its clinical prevention and treatment. Although surgery combined with concurrent radiotherapy and chemotherapy improves the clinical cure rate of early-stage breast cancer, there is a lack of effective treatment for advanced breast cancer [21]. Studies have shown that the benefits of breast cancer chemotherapy are rarely achieved in the subgroup of patients with “highly endocrine-sensitive” tumors, as defined by more than 50% of cells expressing both ER and PR, based on immunohistochemistry, and insufficient amplification of HER-2 [22]. Therefore, there is great interest in exploring the pathogenesis of breast cancer and identifying biological markers for early diagnosis. In recent years, miRNAs have attracted increasing research attention and they have become a new field of tumor biomarker research [18]. Studies have confirmed that *miR-21* [23], *miR-195* [24], and *miR-140-3p* [25] were related to the development of breast cancer.

In the present study, we analyzed the serum levels of three miRNAs, *miR-9-5p*, *miR-17-5p*, and *miR-148a-3p*, in breast cancer cases and healthy controls, and we found that the levels of *miR-9-5p* and *miR-148a-3p* were higher in the case group, whereas no significant difference in the serum levels of *miR-17-5p* was found between the two groups. Furthermore, we evaluated the levels of these three miRNAs according to clinicopathological features of breast cancer and found that the serum levels of *miR-9-5p* were significantly higher in the HER2-positive breast cancer patients compared to the HER2-negative patients. We then performed regression analysis and found that the levels of *miR-9-5p* and *miR-148a-3p* positively correlated with the presence of breast cancer. We further performed specificity and sensitivity analyses to determine their diagnostic value for breast cancer, and we found that the sensitivities of *miR-9-5p* and *miR-148a-3p* were 85.2 (95% CI: 76.3, 99.1) and 86.6 (95% CI: 70.4, 92.8), respectively, and the specificities of *miR-9-5p* and *miR-148a-3p* were 93.7 (95% CI: 72.5, 98.7) and 87.5 (95% CI: 73.8, 93.9), respectively.

In this study, *miR-9-5p* levels were found to be significantly upregulated in the breast cancer patients compared to the healthy controls. Consistent with our findings, Barbano et al. [14] also found elevated levels of *miR-9-5p* in tumor tissues compared to normal breast tissues. *miR-9-5p*, a member of the miR-9 family, is a highly conserved miRNA that is primarily expressed in the central nervous system [26]. The role of *miR-9-5p* in different tumors is inconsistent. It acts as a cancer-promoting miRNA in small cell lung cancer [27] and prostate cancer [28], but as a tumor suppressor in gastric cancer [29] and pancreatic cancer [30]. *miR-9-5p* participates in the proliferation, invasion, tumor metastasis, and angiogenesis of breast cancer through different target genes [31]. Studies have shown that *miR-9-5p* was an important factor affecting the prognosis of breast cancer, and its high expression levels suggest a poor prognosis for patients with breast cancer [32, 33]. Further analysis found that the serum *miR-9-5p* levels were significantly higher in the HER2-positive breast cancer patients compared to the HER2-negative patients, indicating that serum *miR-9-5p* levels may distinguish the HER2-positive subtype of breast cancer. Studies have also found that the expression levels of seven miRNAs (*miR-183*, *miR-660*, *miR-29a*, *miR-93*, *miR-378*, *miR-4281*, and *miR-428*) were significantly different in women with HER2-positive tumors compared to those with HER2-negative tumors [34].

The level of serum *miR-148a-3p* was significantly increased in the patients with breast cancer compared with the healthy controls. Studies have shown that *miR-148a-3p* may be closely related to the occurrence and development of tumors. The expression of *miR-148a-3p* was increased in prostate cancer patients [35], and the downregulation of *miR-148a-3p* in patients with gastric cancer may promote the occurrence of gastric cancer [36].

In thymic epithelial tumors, *miR-148a-3p* is used as a noninvasive marker of treatment efficacy and prognosis [37]. A previous study showed that, by inhibiting the expression of the ER*α* protein, *miR-148a* decreased the viability and migration of breast cancer cells induced by estrogen [38]. Contrary to our results, Li et al. [18] found that *miR-148a-3p* levels were downregulated in the plasma of patients with breast cancer, and they also found that *miR-148a-3p* was associated with lymph node metastasis. These conflicting results may be ascribed to differences in sample size, cancer subtypes, clinical characteristics and methodology, and the difficulties in transferring miRNA-based tests into routine diagnostic applications [39].

*miR-17-5p*, a member of the miR-17-19 family, is involved in a variety of tumorigenic mechanisms. It is related to the proliferation and apoptosis of tumor cells and has been the focus of much research in recent years [40]. Whereas *miR-17-5p* is particularly important in breast cancer, the *miR-17-5p*/*miR-20a* cluster acts as a tumor suppressor to directly inhibit the expression of amplified in breast cancer 1 and cyclin D1 in human breast cancer [41]. Yu et al. [42] found that *miR-17-5p* clusters mediated the migration and infiltration of breast cancer cells. In addition, *miR-17-5p* inhibited the migration and infiltration of MDA-MB-231 cells by inhibiting HBP1 [43]. Some studies have found that the levels of *miR-17-5p* in the serum of breast cancer patients and in breast cancer tissues were lower than those in the serum and tissues of normal control subjects [20, 44]. On the contrary, we found no significant difference in the serum level of *miR-17-5p* in breast cancer patients compared to normal control subjects and *miR-17-5p* levels were poorly correlated with the presence of breast cancer. A possible reason for these contradictory results may be the different disease settings analyzed, as we focused on newly diagnosed breast cancer patients without any prior treatment. Conversely, the study by Wang et al. [20] was conducted among breast cancer patients who had cancer recurrence within 5 years after surgery, and Eichelser et al. [44] analyzed serum samples from 120 patients with primary breast cancer after surgery and before chemotherapy. Another reason for the inconsistent findings may be the small sample size of our study and consequently, the limited statistical power.

However, *miR-9-5p* and *miR-148a-3p*, which have higher sensitivity and specificity than *miR-17-5p*, may have a reasonable diagnostic accuracy for discriminating breast cancer patients from healthy subjects.

Some limitations of this study should be noted. First, sensitivity and specificity were not validated independently. Second, the sample size was relatively small, which limited the overall power of the study. Third, we did not collect follow-up data, such as disease recurrence and survival rates after treatment. Finally, we did not assess the diagnostic efficacy of combined *miRNA-9-5p* and *miR-148a-3*p levels.

## 6. Conclusions

The serum levels of *miR-9-5p* and *miR-148a-3p* were significantly upregulated in breast cancer patients compared with healthy subjects. Thus, *miR-9-5p* and *miR-148a-3p* may be regarded as biomarkers for the clinical diagnosis of breast cancer. However, these results should be confirmed in a larger validation study before these miRNAs can be used for the clinical diagnosis of breast cancer. Moreover, in the future, it will be necessary to evaluate the diagnostic efficacy of the serum levels of these miRNAs in larger, prospective studies that include multiple tumor types. Such data will be necessary to transfer these biomarkers into clinical practice.

## Figures and Tables

**Table 1:** Baseline characteristics of breast cancer patients and controls.

Characteristics	Cases (*n* = 49)	Controls (*n* = 49)	*P*
Age (years)	55.6 ± 9.58	54.2 ± 8.77	0.452
BMI (kg/m^2^)	26.4 ± 5.47	27.3 ± 5.59	0.423
Age of first pregnancy (years)	20.5 ± 2.14	21.0 ± 2.11	0.247
Menopause status			0.655
Premenopause	13 (26.5)	15 (30.6)	
Postmenopause	36 (73.5)	34 (69.4)	
Use of estrogens			0.372
Yes	8 (16.3)	5 (10.2)	
No	41 (83.7)	44 (89.8)	
Family history of breast cancer			0.092
Yes	5 (10.2)	1 (2.0)	
No	44 (89.8)	48 (98.0)	

Abbreviation: BMI: body mass index.

**Table 2:** Clinicopathological characteristics of breast cancer patients.

	*n* (%)
Tumor size (mm)	8.2 ± 5.24
Histology	
IDC	40 (81.6)
ILC	2 (4.1)
Others	7 (14.3)
ER status	
+	30 (61.2)
-	19 (38.8)
PR status	
+	28 (57.1)
-	21 (42.9)
HER2 status	
+	35 (71.4)
-	14 (28.6)

Abbreviations: ER: estrogen receptor; IDC: invasive ductal carcinoma; HER2: human epidermal growth factor receptor 2; ILC: invasive lobular carcinoma; PR: progesterone receptor.

**Table 3:** Serum levels of the three microRNA candidates in the case and control groups.

	Cases (*n* = 49)	Controls (*n* = 49)	*P*
*miR-9-5p*	0.514 ± 0.327	0.356 ± 0.233	0.002
*miR-17-5p*	2.070 ± 0.593	2.177 ± 0.608	0.996
*miR-148a-3p*	2.557 ± 0.891	2.086 ± 0.552	<0.001

Data were expressed as the mean ± standard error.

**Table 4:** Distribution of the levels of the three types of microRNAs by subgroups.

	*miR-9-5p*				*miR-17-5p*				*miR-148a-3p*			
	Cases	Controls	*P*1	*P*2	Cases	Controls	*P*1	*P*2	Cases	Controls	*P*1	*P*2
Menopause status												
Premenopause	0.395 ± 0.312	0.356 ± 0.233	0.587	0.502	2.138 ± 0.716	2.177 ± 0.608	0.844	0.110	2.410 ± 0.784	2.086 ± 0.552	0.092	0.149
Postmenopause	0.557 ± 0.326		0.001		2.045 ± 0.551		0.307		2.609 ± 0.931		0.002	
ER status												
+	0.492 ± 0.329	0.356 ± 0.233	0.035	0.895	2.156 ± 0.621	2.177 ± 0.608	0.883	0.282	2.645 ± 0.810	2.086 ± 0.552	<0.001	0.121
-	0.549 ± 0.330		0.008		1.933 ± 0.533		0.130		2.417 ± 1.014		0.089	
PR status												
+	0.484 ± 0.325	0.356 ± 0.233	0.049	0.944	2.026 ± 0.605	2.177 ± 0.608	0.297	0.380	2.482 ± 0.955	2.086 ± 0.552	0.024	0.224
-	0.563 ± 0.320		0.002		2.128 ± 0.587		0.756		2.656 ± 0.810		0.001	
HER2 status												
+	0.577 ± 0.357	0.356 ± 0.233	0.001	0.049	2.118 ± 0.591	2.177 ± 0.608	0.659	0.797	2.570 ± 0.863	2.086 ± 0.552	0.002	0.481
-	0.357 ± 0.251		0.985		1.949 ± 0.603		0.093		2.524 ± 0.992		0.035	

Abbreviations: ER: estrogen receptor; HER2: human epidermal growth factor receptor 2; PR: progesterone receptor; *P*1: *P* value compared with controls; *P*2: *P* value compared among subgroups of cases.

**Table 5:** Regression analysis of microRNA levels and the presence of breast cancer.

	Regression coefficient	95% CI		*P* value
		Lower limit	Upper limit	
*miR-9-5p*	7.69	5.88	9.79	<0.001
*miR-17-5p*	0.25	1.24	1.88	0.458
*miR-148a-3p*	2.48	0.77	4.20	<0.001

Abbreviation: CI: confidence interval.

**Table 6:** Sensitivity and specificity of serum miRNA levels as markers for distinguishing breast cancer patients from healthy individuals.

	Sensitivity (95% CI)	Specificity (95% CI)
*miR-9-5p*	85.2 (76.3, 99.1)	93.7 (72.5, 98.7)
*miR-17-5p*	70.6 (58.4, 81.93)	65.2 (45.1, 80.7)
*miR-148a-3p*	86.6 (70.4, 92.8)	87.5 (73.8, 93.9)

Abbreviation: CI: confidence interval.

## Data Availability

The data that support the findings of this study are available on request from the corresponding author. The data are not publicly available due to privacy restrictions.

## Abbreviations

BC: Breast cancer
BMI: Body mass index
CI: Confidence interval
ER: Estrogen receptor
HER2: Human epidermal growth factor receptor 2
IDC: Invasive ductal carcinoma
IHC: Immunohistochemistry
ILC: Invasive lobular carcinoma
miRNAs: MicroRNAs
PR: Progesterone receptor
qRT-PCR: Quantitative reverse transcription-polymerase chain reaction
SE: Standard error.
